# Maternal obesity and placental function: impaired maternal–fetal axis

**DOI:** 10.1007/s00404-024-07462-w

**Published:** 2024-03-18

**Authors:** Frank Louwen, Nina-Naomi Kreis, Andreas Ritter, Juping Yuan

**Affiliations:** Obstetrics and Prenatal Medicine, Gynecology and Obstetrics, University Hospital Frankfurt, J. W. Goethe-University, Theodor Stern-Kai 7, 60590 Frankfurt, Germany

**Keywords:** Maternal obesity, Placenta, Placental metabolism, Inflammation, Oxidative stress, Immune cells, Epigenetics

## Abstract

The prevalence of maternal obesity rapidly increases, which represents a major public health concern worldwide. Maternal obesity is characteristic by metabolic dysfunction and chronic inflammation. It is associated with health problems in both mother and offspring. Increasing evidence indicates that the placenta is an axis connecting maternal obesity with poor outcomes in the offspring. In this brief review, we have summarized the current data regarding deregulated placental function in maternal obesity. The data show that maternal obesity induces numerous placental defects, including lipid and glucose metabolism, stress response, inflammation, immune regulation and epigenetics. These placental defects affect each other and result in a stressful intrauterine environment, which transduces and mediates the adverse effects of maternal obesity to the fetus. Further investigations are required to explore the exact molecular alterations in the placenta in maternal obesity, which may pave the way to develop specific interventions for preventing epigenetic and metabolic programming in the fetus.

## Introduction

Obesity, commonly defined by body mass index (BMI), is a growing public health concern and its prevalence is steadily increasing worldwide [1, 2]. It is estimated that 2.7 billion adults will be overweight (BMI 25.0–29.9 kg/m^2^), over one billion will be obese (obesity class I and II, BMI 30.0–39.9 kg/m^2^), and 177 million will be extremely obese by 2025 (obesity class III, BMI ≥ 40.0 kg/m^2^) [3, 4]. While 29.0% of women giving birth had obesity in the United States in 2019 [5], 45.7% of women were overweight or obese in Europe in 2019 [6]. Maternal obesity, characteristic by metabolic dysfunction and chronic inflammation, negatively affects placental function and fetal development [7, 8], resulting in epigenetic and metabolic changes in the offspring [9]. From mother’s perspective, maternal obesity is associated with multiple pregnancy complications, such as spontaneous abortion, Caesarean delivery, and increased risk of developing gestational diabetes mellitus (GDM) and preeclampsia (PE) [10, 11]. Moreover, mothers with obesity are highly associated with hypertension, diabetes, and depression in later life [12]. From infant’s perspective, maternal obesity is linked to small for gestational age (SGA) infants, even more frequently, large for gestational age (LGA) newborns [10, 12], and stillbirth, particularly in males [13]. Importantly, children born to women with obesity are at an increased risk of obesity, metabolic disease, neuropsychiatric and cognitive disorders, and deregulated immunity [14–16]. The association between maternal obesity and health problems in the offspring suggests a transmission of metabolic disease from the mother to the child.

## Maternal obesity

Obesity is caused by an imbalance between food intake and energy expenditure [17]. Obesity affects many systems and organs, such as the adipose tissue, which is an important metabolic and endocrine organ. Adipose tissue produces and releases various bioactive factors, including nutrients, hormones, adipokines, growth factors, enzymes, and extracellular vesicles that modulate energy balance, glucose and lipid homeostasis, tissue repair, inflammatory regulation, and immune response [18–21]. Obesity alters the structure, composition, regulation, and function of adipose tissue, along with many changes in other organs. Maternal obesity is thus associated with deregulated circulating factors, particularly metabolites like glucose and lipids, adipokines including leptin and adiponectin, growth factors for example insulin-like-growth factors (IGFs), and inflammatory cytokines such as interleukin 6 (IL6), IL8, tumor necrosis factor α (TNFα), monocyte chemoattractant protein 1 (MCP-1), and C-reactive protein (CRP) [7, 19, 20]. In particular, elevated lipids, leptin and IL6 are inflammatory mediators, which play important roles in the development of maternal obesity and metabolic dysfunction. While many metabolites in the maternal circulation, such as glucose and lipids, are likely transmitted across the placental barrier, causing fetal hyperglycemia and hyperlipidemia and negatively affecting fetal development [7], an array of maternal metabolic and inflammatory signals directly regulate placental function [22, 23]. Maternal metabolites, hormones, growth factors, and cytokines that are altered in maternal obesity result in an “obesorgenic” metabolic environment, which leads to changes in placental function, fetal growth and development. Although the molecular mechanisms remain elusive, emerging evidence indicates that an impaired placenta, the maternal–fetal axis, mediates this metabolic environment from mothers with obesity to adverse short- and long-term outcomes in the offspring.

## The placenta

The placenta, a temporary organ, is the interface between the mother and the fetus. It is essential for fetal growth and development and a key for a successful pregnancy [24]. The human placenta is composed of a fetal part or chorionic plate and a maternal part or basal plate. The chorionic plate is covered by the amnion, which is composed of a single layer of stratified epithelium and amniotic mesenchyme, an avascular connective tissue [25]. The placenta provides nutrients and oxygen to the growing fetus, produces and releases bioactive factors, such as hormones, growth factors, cytokines, and microRNAs/long non-coding RNAs/circular RNAs (miRNAs/lncRNAs/cirRNAs), and removes waste products [24, 26]. Moreover, the placenta serves as a defense front against pathogens through multiple mechanisms, including triggering interferon type III signaling, miRNA-mediated autophagy and the nuclear factor-κB (NF-κB) pathway [26, 27]. The placenta contains various placental cells including trophoblasts, immune cells, stromal cells and endothelial cells. Its development depends on the differentiation of progenitor cells, termed villous cytotrophoblasts (vCTBs), into the syncytiotrophoblast (STB) as well as extravillous trophoblasts (EVTs) [28]. While the STB builds the important interface between maternal and fetal blood [29], vCTBs of the anchoring villi differentiate into invasive interstitial EVTs, which invade the maternal decidua and remodel the uterine spiral arteries [30]. A variety of molecular signaling pathways, such as Notch and Wnt (wingless/integrated), regulates placental development and controls trophoblast stemness, differentiation, and function [31].

Interestingly, a large body of epidemiological data suggests that altered placental function increases the risk of obesity, metabolic and cardiovascular diseases in the adult life of the offspring [32–34]. In further support, studies in mice demonstrate that the placenta directly impacts fetal brain development and that changed placental function mediates maternal complications to adverse fetal neurodevelopment [35–38]. These observations highlight that the placenta is the key, determining life-long metabolic and mental health and connecting maternal obesity with poor outcomes in the offspring.

## Changed placenta in maternal obesity

Altered bioactive factors in maternal obesity may directly regulate intracellular signaling pathways in trophoblastic cells of the placenta. In line with this notion, the STB, the transporting epithelium in the placenta, expresses receptors for glucose transporters (GLUTs), insulin, leptin, and IGF-1 in its maternal-facing microvillous plasma membrane [39–41]. Through these signaling pathways, maternal obesity associated alterations can thus negatively affect the placenta in diverse aspects, such as placental metabolism, mitochondrial function, inflammation modulation, oxidative response and epigenetics. Pathologically, the rate of maternal placental vascular lesions was higher in women with obesity than in women with normal weight [42]. Maternal obesity was significantly associated with both maternal- and fetal overall vascular malperfusion, inflammatory lesions, and villitis [43]. As placenta is a fetal tissue, it exhibits sexual dimorphism. Indeed, fetal sex affected significantly the effect of maternal obesity on placental inflammatory lesions showing an increased incidence rate of chronic villitis and fetal thrombosis in female placentas [44]. Studies have further revealed distinct sexually dimorphic profiles of gene expression in the placenta, particularly, genes responsible for immune response and inflammatory regulation [45–49]. Early developmental stresses in the placenta are believed to be transduced into the offspring through fetal epigenetic and metabolic reprogramming [9, 50, 51].

### Deregulated metabolism in the placenta

The placenta maintains high metabolic activity to fulfill its roles in providing the fetus with nutrients, hormones and oxygen during pregnancy [52]. While circulating lipids are elevated during pregnancy in all women, maternal obesity is associated with an altered maternal lipid profile: compared with control pregnant women, pregnant women with obesity displayed lower high-density lipoprotein (HDL) levels in the first trimester and higher maternal triglyceride (TG) levels in the second and third trimester [53]. In addition, non-esterified (free) fatty acids (NEFA), hydrolyzed products of TGs, were also elevated in maternal plasma throughout gestation in women with obesity [53].

Altered metabolism in maternal obesity leads to metabolic deregulation in the placenta. Placental omics studies demonstrate that lipid metabolism was altered in the placenta from women with obesity [54–56]. These placentas displayed increased lipoprotein lipase activity [57]. The expression of genes responsible for lipid transport mechanisms was also deregulated, such as genes encoding fatty acid transport protein 2 (FATP2) and FATP4 [58, 59]. This leads to changed placental lipid profile in maternal obesity. In fact, placentas from women with obesity displayed elevated levels of TGs, free fatty acids (FFAs), NEFAs, and cholesterol [8]. The transcriptomic analysis of term placentas from women with obesity further revealed differential expression of genes associated with lipid metabolism, such as decreased *DKK1* (Dickkopf homolog 1) [60] and *ANGPTL4* (angiopoietin-like 4) [61]. In further support, the results from placental proteomic analysis were consistent with increased lipid synthesis and altered antioxidant capacity in placentas from women with obesity [62]. These alterations facilitate placental lipid accumulation, reduce lipid transport to the developing fetus, and induce a lipotoxic placental environment that associates with cellular stress and inflammation. The data strengthen the notion that maternal obesity is associated with placental lipotoxicity [63, 64].

Glucose is the primary substrate for placental and fetal energy metabolism. To promote placental and fetal glucose delivery, pregnancy is accompanied by alterations in maternal glucose metabolism, including insulin resistance, activation of hepatic glucose production and increased β-cell insulin release with higher plasma C-peptide [65]. Women with obesity had 50–60% higher postprandial insulin concentrations than control women in both early and late gestation [66]. Women with obesity were more glucose intolerant than pregnant women with normal weight, as evidenced by higher fasting, 1-h and 2-h glucose levels following an oral glucose tolerance test [66]. Glucose transfer to the fetus occurs via a concentration gradient, which is mainly mediated by the GLUT family. The expression of different isoforms of the GLUT transporter was altered by an obesogenic maternal environment and these alterations mirrored the trends in fetal growth and birth weight at term [67, 68]. These findings underscore the notion that deregulated metabolism of maternal obesity leads to deregulated placental metabolism.

### Altered immune cells in the placenta

Maternal immunity plays a critical role in pregnancy and the development of healthy offspring. Maternal immune cells, including uterine macrophages, natural killer cells, dendritic cells and mast cells, are present in the placenta [69]. While maintaining host defense against pathogens [26], maternal immune cells initiate and support implantation, placentation, and parturition [70]. Maternal obesity enhanced the number of maternal macrophages and innate immune cells associated with accumulated macrophages in the placental villous stroma, promoting inflammation, oxidative stress, mitochondrial dysfunction and metabolic deregulation [71, 72]. In addition, an increased ratio of pro-inflammatory M1 macrophages versus anti-inflammatory M2 macrophages was reported [73]. These macrophages produced pro-inflammatory cytokines, including IL6, TNFα and MCP-1 [71, 74, 75], enforcing placental inflammation. Moreover, the placenta normally helps to skew the maternal and fetal environment toward a CD4^+^ helper T cell type-2 (Th2) and anti-inflammatory profile, whereas obesity and other stress factors create a more CD4 ^+^ helper T cell type-1 (Th1) and inflammatory gestational environment [76]. Furthermore, maternal obesity and obesogenic diets have been associated with abnormal immune function in the offspring, including decreased response to infection, atopic disease, and asthma [77–79].

### Inflammatory placenta

Obesity is associated with enhanced inflammation, referred to as “metaflammation” for the chronic, low-grade inflammatory state [80]. Metaflammation is triggered by metabolites and nutrients that lead to systemic insulin resistance [80, 81]. This metaflammation in pregnant women with obesity initiates a cascade of events, leading to an inflammatory utero environment.

Indeed, maternal cytokines and adipokines link maternal metaflammation to placental function. Similar to maternal plasma, the placenta also showed increased levels of inflammatory markers, such as IL6, IL8, IL1β, and MCP-1 [81, 82]. Multiple factors, including lipids [83], oxidized lipids [84], reactive oxygen species (ROS) [85], and endotoxin [86], stimulate placental inflammation. This cytokine profile is driven by multiple inflammatory pathways, including the activation of receptors for advanced glycation end products (RAGEs) [87] and activation of Toll-like receptor 4 (TLR4) [88]. In turn, these pathways promote NF-κB, c-Jun N-terminal kinase (JNK), and rat sarcoma virus (Ras) signaling, resulting in an increased generation of ROS and secretion of the inflammatory cytokines [89]. Placental inflammation impairs overall placental function. Particularly, early inflammation has been reported to affect the developing immunophenotypes of fetal immune cells, which is likely related to the effects of obesity on epigenetics and the microbiome [77, 90, 91].

### Stressed placenta

Pregnancy is linked to heightened oxidative stress, partially due to the high metabolic demand of the placenta [90]. Mitochondria are the major source of ROS under normal physiological conditions. ROS are vital signaling molecules of redox-sensitive pathways, including autophagy, cell differentiation, and inflammatory response [92]. ROS triggered the expression of vascular endothelial growth factor (VEGF) and GLUTs to promote angiogenesis in early pregnancy [93]. In contrast, excess ROS generated by mitochondria and/or decreased total antioxidant capacity (TAC) were shown to disrupt cellular and tissue homeostasis by promoting oxidative stress, damaging proteins, lipids, and nucleic acids [8]. Maternal obesity was associated with increased maternal ROS, including higher levels of maternal nitric oxide and superoxide anions [94, 95]. Moreover, ROS production [72], glutathione concentrations and superoxide dismutase (SOD) activity [95] in the placenta were reported to be increased in maternal obesity, which may impair mitochondrial function and reduce ATP production [72]. In further support, the proteomic signature showed altered antioxidant capacity in placentas from women with obesity [62]. Reduced placental mTOR gene expression and up-regulation of genes involved in oxidative stress and mitochondrial function, such as increased sirtuin 1 (*SIRT1*) and uncoupling protein 2 (*UCP2*), were reported in maternal obesity [96]. It was also revealed that highly increased ROS led to mitochondrial dysfunction, placental inflammation and fetal epigenetic changes [97, 98].

Inflammation and metabolic dysfunction also increase placental endoplasmic reticulum (ER) stress and downstream activation of the placental unfolded protein response (UPR), which has been extensively reviewed [8]. Along with deregulated metabolism, inflammation, immune deregulation, these cellular stresses impair placental function and fetal development, which may cause long-term alterations in the immune and nervous system of the offspring [70].

### Placental epigenetic changes

Altered placental epigenetics, including DNA methylation, may mediate adverse outcomes in the offspring [50]. Compared to a plenty of placental epigenetic investigations in pregnancy complicated by diabetes, only a smaller proportion of studies focused on epigenetic alterations in pregnancy complicated by obesity alone [50]. Nevertheless, differentially-methylated genes, such as *ADIPOQ* (adiponectin), *ADIPOR1* (adiponectin receptor 1), *LEP* (leptin) and *LEPR* (leptin receptor), were reported in placental tissue in maternal obesity [99, 100]. Recently, it has been revealed that placental DNA methylation alterations were associated with maternal pre-pregnancy BMI and gestational weight gain [101]. Maternal obesity is further reported to be linked to increased DNA methylation and decreased RNA methylation in the human term placenta [102]. Interestingly, based on the data derived from ten studies with 2631 mother-child pairs from the Pregnancy and Childhood Epigenetics (PACE) consortium, 27 CpG sites were identified to be differentially methylated in placental tissue DNA from women with obesity [103]. Moreover, 104 CpG sites annotating for 97 genes in the placenta were reported to be differentially methylated with gestational weight gain [104]. Particularly, CpG sites annotating for *FRAT1* (frequently rearranged in advanced T cell lymphomas-1), *SNX5* (sorting nexin 5) and *KCNK3* (potassium channel subfamily K member 3) genes were correlated with an adverse metabolic phenotype in the offspring [104]. In sum, these data demonstrate that maternal obesity is associated with epigenetic changes in the placenta. More studies are needed to further explore the impact of maternal obesity on epigenetic alterations in placental tissue as well as in various placental cell populations at different gestational stages.

### Cord blood cell epigenetic alterations

Placental dysfunctions associated with maternal obesity affect each other, resulting in a stressful intrauterine environment, which associates with poor outcomes, especially, with programing the fetus for disease in later life [47, 105, 106]. Indeed, maternal pre-pregnancy BMI was linked to decreased methylation at five CpG sites near the *LEP* transcription start suggesting an association between maternal and fetal obesity [107]. Methylation of serotonin regulating genes in cord blood cells was correlated with maternal metabolic parameters [108]. Moreover, gestational weight gain in pregnant women with obesity was associated with cord blood cell DNA methylation [109]. Average methylated cytosine levels in both the CpG islands and promoters were shown to be significantly decreased in cord blood from overweight and obese groups [110]. Importantly, a longitudinal birth cohort study, which was across a period from birth to 18 years, showed a significant connection between cord DNA methylation marks and postnatal BMI trajectories [111]. These data show that fetal epigenetic alteration is a potential underlying mechanism for poor outcomes of the offspring.

### Potential clinical intervention

Restoration of placental function will reduce the adverse outcomes caused by maternal obesity. Prior and during pregnancy are time windows to prevent the negative consequences of poor in utero environments and to improve the long-term outcomes of the mother and the child. It is necessary for women of reproductive age to receive education about maternal and fetal risks associated with maternal obesity. Exercise and lifestyle modifications may positively affect maternal and fetal outcomes. In fact, exercise and healthy diets during pregnancy were shown to be able to influence the offspring’s lean mass and early growth [112]. Further potential interventions, including supplementation of omega 3 polyunsaturated fatty acids (n-3 LCPUFAs), DHA (docosahexaenoic acid), melatonin, or anti-inflammatory agents, have been discussed [7]. Activation of the adiponectin receptor in the placenta has also been proposed to be a promising strategy [7]. This is supported by the data from animal experiments showing that normalization of maternal adiponectin in obese pregnant mice prevented cardiac dysfunction and improved glucose metabolism in the adult offspring [113, 114]. Moreover, studies have underlined the importance of the gut microbiome in the transmission of the obesity phenotype and dietary interventions are thus considered as potential strategy to improve maternal and fetal outcomes [115–117]. Especially, novel anti-inflammatory diets during pregnancy should be explored to prevent metabolic dysfunction in the offspring [118]. In addition, vitamin D deficiency has been reported to be partially responsible for placental mitochondrial dysfunction and increased inflammation, and its supplementation is thus proposed to be beneficial in improving placental function [119]. Collectively, although much has been done, it is still a long way to go to discover targeted and effective strategies to prevent and reduce adverse maternal and fetal outcomes induced by maternal obesity.

## Conclusion

**Fig. 1:**
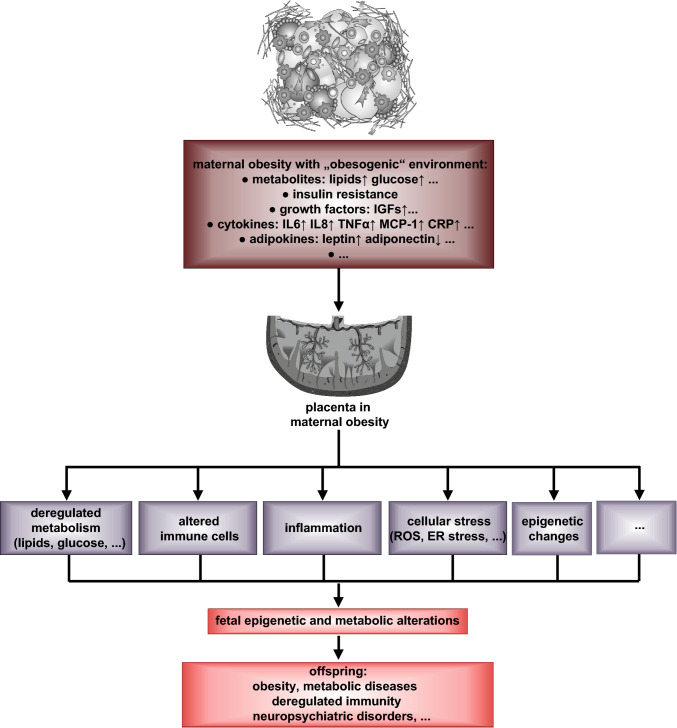
Schematic illustration showing the placenta as an axis linking maternal obesity to poor outcomes in the offspring. Maternal obesity, associated with deregulated metabolism and inflammation, negatively affects placental development and function, evidenced by defected metabolism, deregulated immune cells, inflammation, cellular stress, changed epigenetics and other unknown aspects. These placental defects affect each other and cause a stressful intrauterine environment, which transduces the effect of maternal obesity to fetal development, leading to poor outcomes in the offspring. *IGF* insulin-like growth factor, *IL* interleukin, *TNFα* tumor necrosis factor α, *MCP-1* monocyte chemoattractant protein 1, *CRP* C-reactive protein, *ROS* reactive oxygen species, *ER* endoplasmic reticulum

The prevalence of maternal obesity is rapidly increasing and the poor short- and long-term outcomes in both mothers and infants represent a major public health problem worldwide. In this brief review, we have summarized the data showing that maternal obesity associated with deregulated metabolism and metaflammation greatly impairs placental development and function, as evidenced by placental defects in lipid and glucose metabolism, stress response, inflammation, immune regulation and epigenetics (Fig. 1). These defects affect each other and result in a stressful intrauterine environment, which transduces and mediates the adverse effects of maternal obesity to the fetus, leading to poor outcomes in the offspring (Fig. 1).

The placenta holds the key to better understand the molecular pathophysiology linking maternal obesity to poor outcomes. Further investigations are required to explore molecular alterations in the placenta in response to maternal obesity. In particular, advanced sequencing approaches [120, 121] represent powerful tools to further study placental ‘omics’ in maternal obesity. The establishment of human trophoblast stem cells [122] and placental organoids [123, 124] also provides novel tools for investigating the impact of maternal obesity on placental function. In addition to trophoblasts, placental mesenchymal stromal/stem cells [125] may also play important roles in mediating the effect of maternal obesity on the placenta. Studies employing these novel techniques may pave the way for developing specific interventions to prevent epigenetic and metabolic programming in the offspring.

## Data Availability

Not applicable.
